# Tramadol‐induced intracerebral hemorrhage: A rare case report

**DOI:** 10.1002/ccr3.7205

**Published:** 2023-04-12

**Authors:** Nor Osman Sidow, Mohamed Farah Osman, Mohamed Sheikh Hassan, Abdulkadir Ahmed, Abdiwahid Ahmed Ibrahim

**Affiliations:** ^1^ Department of Neurology Mogadishu Somali – Turkey Recep Tayyip Erdoğan Training and Research Hospital Mogadishu Somalia

**Keywords:** basal ganglia, hemorrhagic stroke, tramadol

## Abstract

A previously healthy middle‐aged man with no vascular risk factors developed a hemorrhagic stroke. He had been using tramadol due to erectile dysfunction for 2 years. So, the patient developed right basal ganglia due to tramadol addiction.

## 1 INTRODUCTION

Tramadol is a centrally acting synthetic analgesic, chemically related to morphine and codeine. Tramadol abuse is being used by many people with different adverse effects, most likely seizures and respiratory depression. Some men use tramadol for erectile dysfunction. We report a case of tramadol‐induced intracerebral hemorrhage. A 36‐year‐old man with no history of chronic diseases presented to the emergency department with a sudden onset of left‐sided weakness. He had no other conventional vascular risk factors such as hypertension, diabetes, or dyslipidemia. A neurological examination showed right gaze preference and mild right facial droop with left side hemiplegia, and the left Babinski sign was positive. He denied smoking cigars or consuming khat, and he had no family history of stroke. Routine vital signs were normal. The electrocardiogram was normal. And routine blood investigations were normal. NIH Stroke Scale = 22 when he came in. A CT scan showed a 53 × 24‐mm hemorrhage in the right basal ganglia region. He had an MRI angiogram, which showed normal intracranial and extracranial vessels. The patient confirmed that he had been using tramadol for 2 years due to erectile dysfunction. A previously healthy middle‐aged man with no vascular risk factors developed a hemorrhagic stroke. It was reported that he had been using tramadol for 2 years. So, the patient developed the right basal ganglia due to tramadol addiction. It is the first time in the literature that tramadol‐induced intracerebral hemorrhage has been reported.

Tramadol hydrochloride (Ultram; OrthoMcNeil Pharmaceutical, Inc.) is a centrally acting synthetic analgesic. Tramadol was authorized for sale as a secure analgesic in 1995 under the brand “Ultram®.” Initially, recent research has shown that the main contributor to its pharmacological efficacy is its opioid activity. Many doctors feel comfortable prescribing it to drug abusers who are in recovery from addiction and to those who are already abusing drugs because the product's labeling is inadequate and its misuse potential has not been shown. Because of this, there have been many cases of abuse and dependency.^1^, ^2^ Tramadol toxicity has been associated with multiple conditions, such as seizure, addiction but not as much as causing stroke.

The relationship between tramadol and sexual function might be thought of as dialectical. On the contrary, there is evidence to suggest that men who suffer from premature ejaculation (PE) may benefit from the unapproved use of tramadol.^3^ Here, we report a case of intracerebral hemorrhage due to tramadol addiction. This is the first time in the literature that tramadol has been said to cause a hemorrhagic stroke.

## 2 CASE REPORT

A 36‐year‐old man with no history of chronic diseases presented to the emergency department with a sudden onset of left‐sided weakness. He had no speech or visual disturbances, no headache, no vomiting, or dizziness. No seizure had been witnessed. He had no other conventional vascular risk factors such as hypertension, diabetes, or dyslipidemia. On examination, normal observations were made. His pulse was regular, his chest was clear, and his heart sounds were normal. Blood glucose was 102 mg/dL, and blood pressure was 130/81. His abdomen was soft and nonsensitive, and his calves were neither swollen nor tender. A neurological examination showed that the cranial nerves were normal except for a right gaze preference and mild right facial droop. Motor examination: 0/5 power in the proximal and distal parts of the left upper and lower limbs. He had an upward left plantar reflex (the Babinski sign is positive). His sensation was normal.

**FIGURE 1:**
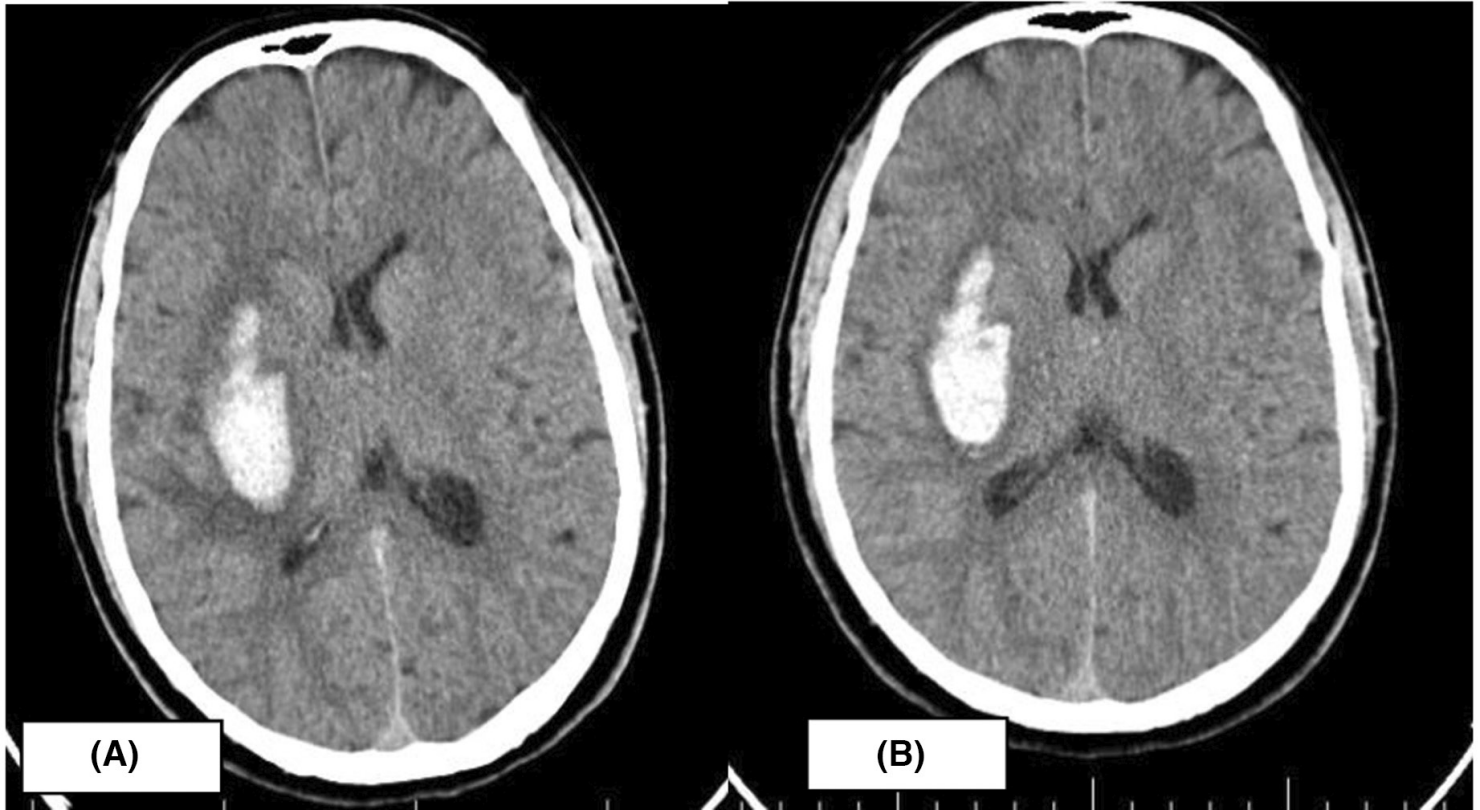
CT head showing hematoma of 53 × 24 mm in the right basal ganglia.

The patient had a normal ECG and a score of 22 on the National Institutes of Health Stroke Scale (NIH Stroke Scale = 22) when he came in. Routine blood examination: hemogram, renal, and liver function tests were normal; electrolytes and coagulation profiles also were normal. A head CT (Figure 1A,B) showed a 53 × 24 mm hemorrhage in the right basal ganglia region.

He had an MRI angiogram, which showed normal intracranial and extracranial vessels. The patient was diagnosed with a hemorrhagic stroke and admitted to the neurology department. Treatment of antibrain edema was started with regular control of blood pressure. The patient started to develop agitation. After asking for a complete history of drug addiction, the patient confirmed that he had been using tramadol for 2 years due to erectile dysfunction. Throughout, he was hemodynamically stable, with a systolic blood pressure of between 110 mm Hg and 130 mm Hg and a heart rate of between 86 beats per minute and 95 beats per minute; the diastolic pressure was normal. For the agitation, we started haloperidol 5 mg injections twice a day intramuscularly, with significant improvement from the agitation.

**FIGURE 2:**
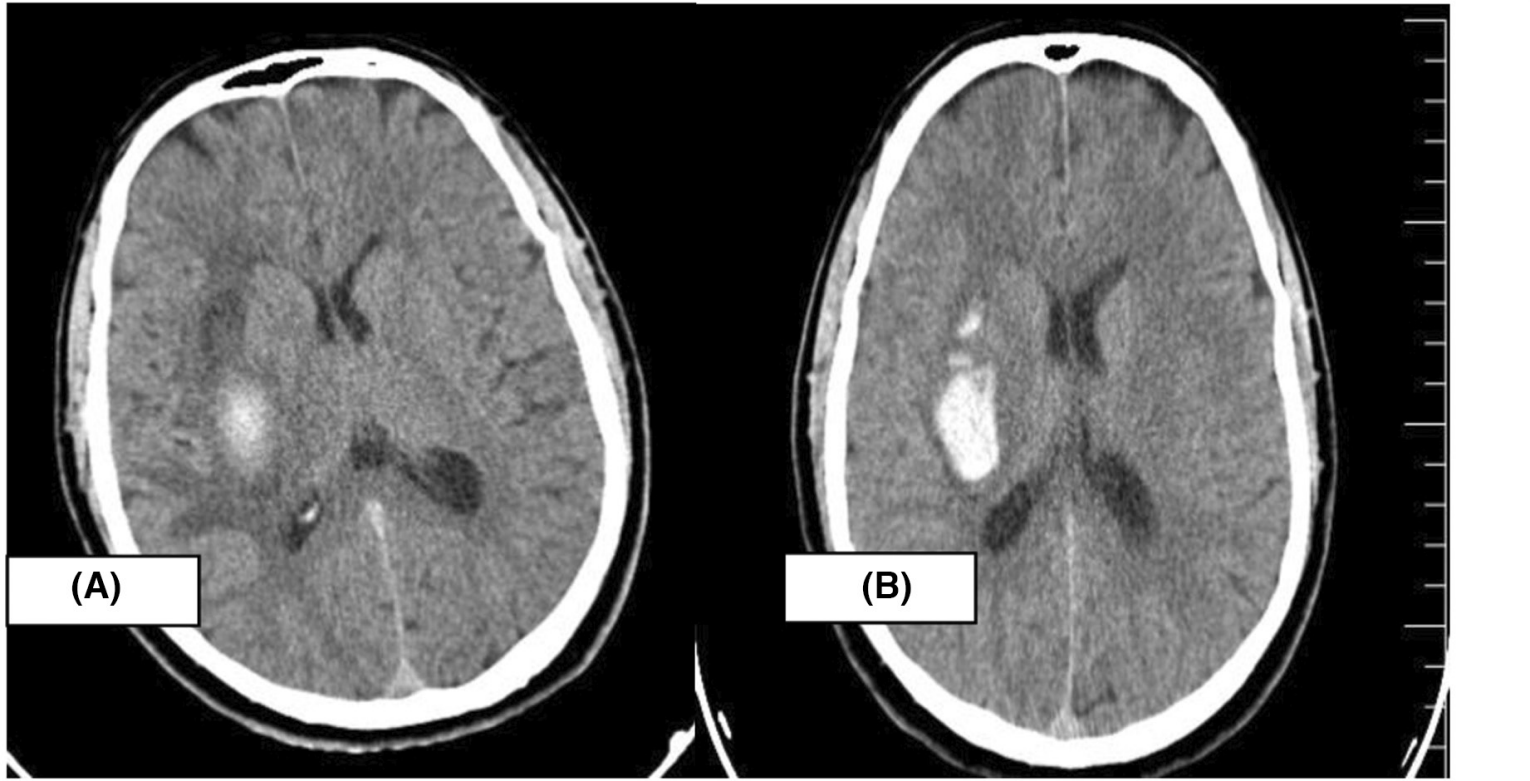
CT head showing slight hematoma resorption of 51 × 22 mm in the right basal ganglia after 5 days.

**FIGURE 3:**
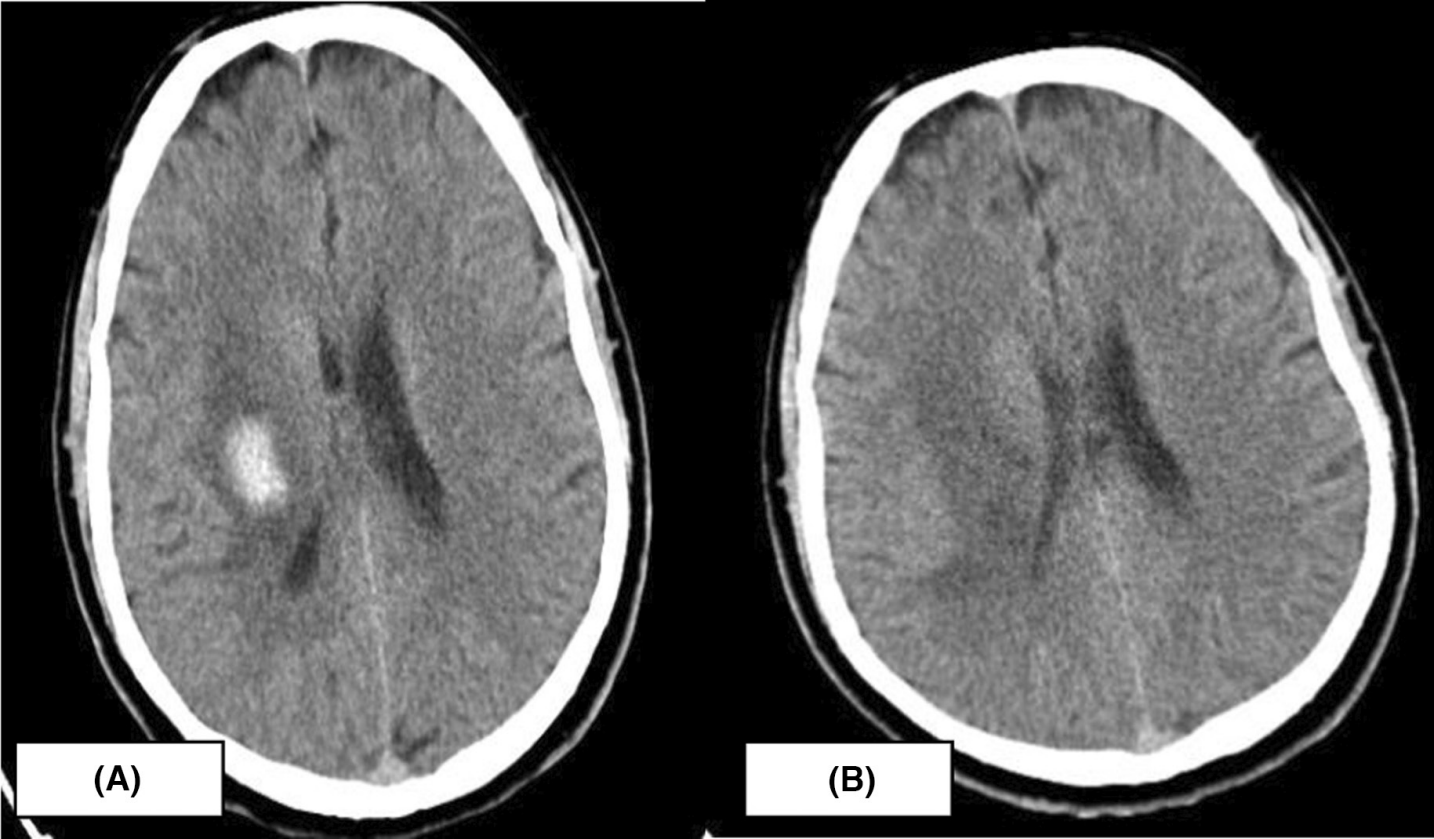
CT head showing significant resorption of the hematoma **(**12 × 24 mm) after 20 days since admission.

So we decided to discharge the patient after 1 week of stabilization and did a control head CT (Figure 2A,B) before discharge, and it showed slight resorption of the hematoma in the right basal ganglia with vasogenic edema. After 20 days, we repeated the CT head (Figure 3A,B) and showed significant resorption of the hematoma **(**12 × 24 mm) with improvement of the left side (power 2/5).

## 3 DISCUSSION

The two enantiomers of tramadol, a centrally acting analgesic chemically related to morphine and codeine, each contribute to analgesic efficacy in a unique way. Both (+)‐Tramadol and its byproduct (+)‐O‐desmethyl‐Tramadol (M1) are opioid receptor agonists. Tramadol blocks the reuptake of norepinephrine and serotonin, which slows down the way the spinal cord sends pain signals.^5^ Results of the retrospective cohort analysis revealed that less than 1% of all tramadol users in the general population experienced a presumed incident seizure occurrence, as indicated by claims data.^4^

Additionally, confusion, drowsiness, convulsions, and respiratory depression are negative consequences of tramadol misuse. Any of these bad effects makes it more likely that a driver, passenger, or pedestrian will be in an accident because it affects performance and good judgment. Numerous case reports have been reported to cause seizures in nonepileptic people. Tramadol use increases mortality and the risk of major adverse cardiovascular events in rheumatoid arthritis patients.^5^

It has been demonstrated that higher doses of tramadol (200 and 400 mg), but not lower doses (50 and 100 mg), also chronic oral tramadol intake, even in the therapeutic concentrations (100–300 ng/mL) for a dose of tramadol, which is always prescribed in treatment of mild‐to‐moderate pain, may lead to physical dependence, boost its effects, and dose‐dependent opioid like withdrawal symptoms. However, lower doses of tramadol (50 and 100) Because of this, many nations throughout the world have placed tramadol on their lists of substances that are subject to controlled distribution.^6^, ^7^ There is a theory that long‐term tramadol addiction raises blood pressure and eventually causes small arteries in the brain to rupture, resulting in hemorrhagic stroke, but there is not enough evidence to back it up. There have been a few rare case reports of strokes with different etiologies, such as strokes of unknown origin and abnormal behavior associated with hemorrhagic stroke.^6^, ^8^, ^9^

Our case was that of a middle‐aged man who had been using tramadol for 2 years for erectile dysfunction. He used a dose of 200–300 mg per day, as he said he benefited from using it, and he spent more time in bed as reported. So he became addicted to it and unable to hold it. He had no history of chronic diseases such as hypertension, diabetes, or hyperlipidemia. He denied smoking or using khat (Catha edulis), which is a plant that is deeply rooted in the cultural life of East African and southwestern Arabian populations. His blood pressure during the presentation and routine monitoring was normal. So, this is the first time to report for tramadol‐induced intracerebral hemorrhage in the literature. There have been reports of seizures and addiction to tramadol, but no reports of strokes yet.

On the MRI brain and angiogram, there were no intracranial and extracranial vessel abnormalities such as aneurysms, cavernomas, or masses. Only the CT scan revealed a hematoma in the right basal ganglia. After few control of the CT head, there was significant resorption of the hematoma and with gradually recovering from the weakness in the left side, the patient started to walk with no assistance.

## 4 CONCLUSION

A previously healthy middle‐aged man with no vascular risk factors developed a hemorrhagic stroke. He had been using tramadol due to erectile dysfunction for 2 years. So, the patient developed right basal ganglia due to tramadol addiction. All the stroke workups did not show an etiology, the only found was the drug addiction of tramadol.

There is a theory that long‐term tramadol addiction raises blood pressure and eventually causes small arteries in the brain to rupture, resulting in hemorrhagic stroke, but there is not enough evidence to back it up. It is the first time in the literature to report tramadol‐induced intracerebral hemorrhage.

## AUTHOR CONTRIBUTIONS

**Nor Osman Sidow:** Conceptualization; project administration; supervision; writing – original draft; writing – review and editing. **Mohamed Farah Osman:** Conceptualization; investigation. **Mohamed Sheikh Hassan:** Data curation; methodology. **Abdulkadir Ahmed:** Validation; visualization. **Abdiwahid Ahmed Ibrahim:** Formal analysis; software.

## FUNDING INFORMATION

None.

## CONFLICT OF INTEREST STATEMENT

The authors declare no conflict of interest.

## ETHICAL APPROVAL

There is no need ethical approval for the case repots in our hospital.

## CONSENT

Written informed consent was obtained from the patient for publication of this case report and accompanying images. A copy of the written consent is available for review by the Editor‐in‐Chief of this journal on request.

## GUARANTOR

Nor Osman Sidow, the corresponding author.

## Data Availability

The data are available for the corresponding author if needed or the Editor‐in‐Chief.
